# The Beliefs About Breastfeeding Questionnaire (BAB‐Q): A psychometric validation study

**DOI:** 10.1111/bjhp.12498

**Published:** 2020-12-19

**Authors:** Philippa Davie, Debra Bick, Joseph Chilcot

**Affiliations:** ^1^ Health Psychology Section Department of Psychology Institute of Psychiatry, Psychology and Neuroscience Guy’s Hospital King’s College London UK; ^2^ Professor of Clinical Trials in Maternal Health Warwick Medical School Warwick Clinical Trials Unit University of Warwick UK; ^3^ Department of Women and Children’s Health Faculty of Life Sciences and Medicine St Thomas’ Hospital King’s College London UK

**Keywords:** beliefs, breastfeeding, breastfeeding experience, health behaviour, psychometric validation, questionnaire

## Abstract

**Objectives:**

Questionnaires used to assess women’s beliefs as a predictor of breastfeeding behaviour are not theoretically informed or tested for psychometric validity and reliability. This study conducted a psychometric evaluation of the Beliefs About Breastfeeding Questionnaire (BAB‐Q).

**Design:**

A two‐phase evaluation in an online cross‐sectional questionnaire study (*N* = 278) and cohort study sample (*N* = 264). A ten‐item questionnaire was proposed to assess women’s beliefs about the benefits and efforts of breastfeeding.

**Methods:**

Exploratory factor analysis (EFA) and confirmatory factor analysis (CFA) assessed construct validity and reliability. Multivariate regression analyses assessed validity in predicting breastfeeding behaviour and experiences.

**Results:**

EFA found a shortened 8‐item, 2‐factor model had good fit (χ^2^ = 23.3, df = 13, *p* < .040; CFI = .99, TLI = .99, RMSEA = .05), with significant factor loadings. Factor 1 (benefit beliefs) and factor 2 (effort beliefs) accounted for 47 and 19.4% of the explained variance and correlated moderately (*r* = −.40). CFA confirmed the solution in the cohort sample (χ^2^ = 49.6 df = 19, *p* < .010; CFI = .97, TLI = .96, and RMSEA = .078). Adjusted regression analyses found beliefs did not reliably predict infant feeding practices. Women’s beliefs significantly predicted the likelihood that women experienced breastfeeding as ‘much more’ positive and negative than they expected.

**Conclusions:**

The eight‐item questionnaire showed good model fit with acceptable loadings, and good reliability for all subscales. The utility of the BAB‐Q at predicting breastfeeding behaviour remains unclear and unsupported by empirical evidence. Further assessments of the predictive validity of the questionnaire in longitudinal studies with diverse beliefs and infant feeding practices are required.

**Statement of contribution**

***What is already known on this subject?***

- Breastfeeding is a public health priority, but breastfeeding rates remain low worldwide
- Psychosocial factors associated with breastfeeding behaviour provide targets for support interventions
- Breastfeeding belief measures are not currently psychometrically valid, reliable, or theoretically informed

***What does this study add?***

- Psychometric evaluation of a breastfeeding belief questionnaire shows it is reliable with good construct validity
- The questionnaire predicted women’s breastfeeding experiences that were ‘more positive’ or ‘more negative’ than expected
- Predictive validity of the questionnaire for breastfeeding behaviour was unsupported by empirical evidence

## Background

The World Health Organization (WHO) (2013) recommends women exclusively breastfeed their infants for the first 6 months of life and encourages extended breastfeeding up to 2 years. Breastmilk is considered to be the optimal method of human nutrition, providing life‐course health benefits for women and infants (Horta, Bahl, Martines, & Victora, 2007; Victora et al., 2016). Despite guidelines to support breastfeeding (WHO, 1998; WHO, 2018a), breastfeeding rates rapidly decline across the postpartum period, and rates worldwide remain low, with <40% of infants being exclusively breastfed for the first 6 months (Victora et al., 2016). There are recognized factors that facilitate the uptake and continuation of breastfeeding; however, many are non‐modifiable demographic attributes. To identify malleable targets for breastfeeding promotion intervention, research has frequently used social‐cognition models to understand factors associated with infant feeding.

Evidence shows that stronger intentions for breastfeeding (Lawton, Ashley, Dawson, Waiblinger, & Conner, 2012; Martinez‐Brockman, Shebl, Harari, & Perez‐Escamilla, 2017), stronger breastfeeding self‐efficacy (DeJager et al., 2015; Dodgson, Henly, Duckett, & Tarrant, 2003; McQueen, Sieswerda, Montelpare, & Dennis, 2015), and positive breastfeeding attitudes (McMillan et al., 2008; Scott, Binns, Oddy, & Graham, 2006; Zhu, Zhang, Ling, & Wan, 2017) are associated with increased initiation rates and longer breastfeeding durations. Evidence also suggests that negative attitudes to formula feeding (Richetin, Conner, & Perugini, 2011) and greater ‘faith in breastmilk’ (O’Brien, Buikstra, & Hegney, 2008) predict breastfeeding behaviour, whereas positive beliefs about formula (Swanson & Power, 2005), more vicarious experience of formula feeding (Bartle & Harvey, 2017), and greater fears of inadequate nutrition (Shepheard, Walbey, & Lovell, 2017) significantly predict formula‐feeding behaviour. Psychometrically validated questionnaires are available and commonly used to assess breastfeeding attitudes and self‐efficacy; however, women’s beliefs about breastfeeding are somewhat neglected as a psychosocial predictor of behaviour (Lewallen, 2006). Where research has measured women’s beliefs, measures have not been psychometrically validated.

Social‐cognition theories of health behaviour fundamentally assume individuals develop beliefs that affect the interpretation of information and guide behaviour (Connor & Norman, 2005). In the absence of validated questionnaires, infant feeding research has frequently relied on measures of attitudes (Lou et al., 2014; Scott et al., 2006; Zhu et al., 2017) to capture women’s cognitive representations of infant feeding. The Iowa Infant Feeding Attitude Scale (IIFAS) (De La Mora, Russell, Dungy, Losch, & Dusdieker, 1999) is a widely used validated scale, but the scale assumes positive attitudes to formula feeding and breastfeeding are antithetical. The IIFAS captures a total score with lower scores indicating positive attitudes to formula feeding and higher scores reflecting positive attitudes towards breastfeeding. Behaviourally, formula feeding and breastfeeding are antagonistic, given there is no other suitable alternative for infant nutrition. However, with respect to psychological appraisal, cognitive representations of infant feeding practices are not antagonistic, and positive (or negative) appraisals of the behaviours are not mutually exclusive. Women can have positive representations about formula feeding and breastfeeding simultaneously, and empirical evidence suggests attitudes for breastfeeding and formula feeding predict infant feeding practices independently (Richetin et al., 2011). Furthermore, women’s motivations for infant feeding are shown to be distinct for breastfeeding and formula feeding (Arora, McJunkin, Wehrer, & Kuhn, 2000). This suggests that women’s beliefs about breastfeeding should be assessed independently of beliefs about formula feeding to accurately predict likelihood of breastfeeding behaviour.

Beliefs towards breastfeeding (Bartle & Harvey, 2017; Swanson, Hannula, Eriksson, Wallin, & Strutton, 2017; Swanson & Power, 2005) have been assessed using items based on UK consensus survey data from 1975 (Manstead, Proffitt, & Smart, 1983). Other studies have measured breastfeeding beliefs using scales specifically developed for use in secondary school girls, but the origin of the questionnaire remains unknown (Humphreys, Thompson, & Miner, 1998; Kloeblen, Thompson, & Miner, 1999). Semenic et al. (2008) used belief items ‘taken from a combination of survey reported in the literature’ (Martens & Young, 1997). More recent research (O’Brein et al., 2008; Shepheard et al., 2017) has used individual questionnaire items, or a selection of items from multiple questionnaires (Lou et al., 2014) to assess women’s beliefs. The Maternal Breastfeeding Evaluation Scale (Leff, Jefferis, & Gagne, 1994) was developed through qualitative interviews, expert panel ratings, and factor analysis, although it includes some outdated items which may not be appropriate in the current socio‐cultural context of breastfeeding (e.g., ‘Breastfeeding made me feel like a cow’ and ‘Breastfeeding made me feel like a good mother’). The questionnaire is also 30 items long, which may be too long for use in clinical or health education contexts, and is designed to capture women’s beliefs during the period of breastfeeding, limiting its application as an antenatal evaluation tool. Taken together, many items used in past studies are not theoretically informed, have not been psychometrically tested, and require updating for use in the context of breastfeeding in the 21st century, which acknowledges breastfeeding as both challenging and rewarding.

Qualitative research has highlighted that in spite of strong motivations or intentions for breastfeeding, women do not always appraise breastfeeding as an inherently positive experience describing breastfeeding as challenging, ‘a maternal duty’, exhausting, and difficult at the same time as being an enjoyable, loving, and fulfilling experience (Brown & Lee, 2011; Lagan, Symon, Dalzell, & Whitford, 2014; Marshall, Godfrey, & Renfrew, 2007; Murphy, 2000; Sheehan, Schmeid, & Barclay, 2013; Shloim et al., 2015). Measurements assessing women’s cognitive representations of breastfeeding have largely neglected this paradox in beliefs and behaviours. Including both positive and negative attributes of breastfeeding, as in the Maternal Breastfeeding Evaluation Scale (Leff et al., 1994), and conceptualizing breastfeeding as a health behaviour that functions on an implicit ‘cost‐benefit’ analysis (where women will weigh the perceived or experienced benefits of breastfeeding against the costs or efforts incurred) (Racine, Frick, Guthrie, & Strobino, 2009) may improve the explanatory power of questionnaires seeking to assess women’s beliefs to predict infant feeding behaviour.

Social‐cognition theories assume beliefs are susceptible to health education (Connor & Norman, 2005), and as such, support strategies for breastfeeding continue to focus on antenatal education to highlight the benefits of breastfeeding (NICE, 2019; WHO, 2018a) and promote breastfeeding as best practice. As support strategies aim to focus on tailoring infant feeding education and support for women (WHO, 2018b), investigating beliefs associated with breastfeeding practices using psychometrically validated measurement tools could provide direction for theoretically informed individual‐level breastfeeding support interventions, which are currently lacking (Davie et al., 2019). This study therefore aimed to carry out a psychometric evaluation of the Beliefs About Breastfeeding Questionnaire (BAB‐Q) and explore the utility of the questionnaire in predicting early infant feeding practices and experiences.

## Methodology

### Design

Two study samples were used; an online cross‐sectional questionnaire study (*n* = 278) analysed the BAB‐Q using exploratory factor analysis (EFA). A confirmatory factor analysis (CFA) was then used to evaluate the BAB‐Q in a cohort study sample (*n* = 264).

### Participants and procedure

#### Online sample

Women were eligible for the online questionnaire study if they reported to be at least 18 years old, and had given birth to a healthy, term (>37 + 0 weeks of gestation), single infant in the last 90 days who was not admitted to neonatal care. Only women living in the United Kingdom (UK) were eligible. Women were recruited online on an opportunity basis between 18 December 2017 and 01 May 2018 using JISC Online Surveys. Study advertisements were displayed both online (i.e., social media) and in paper (i.e., on local public notice boards). Women were recruited by accessing the online link provided in advertisements. A study information page, consent form, and eligibility questionnaire were read and signed prior to questionnaire completion. The questionnaire contained three sections that collected data on women’s socio‐demographic background and general health (‘About You’), women’s infants (‘About Your Baby’), and ‘About Infant Feeding’: infant feeding practices, previous feeding experience, and the BAB‐Q. At questionnaire completion, women were offered the chance to take part in a prize draw to win a shopping voucher, as thanks for their contribution.

#### Cohort sample

Women were recruited in‐person antenatally (≥28 + 0 weeks of gestation) as part of a prospective longitudinal cohort study, according to eligibility criteria described above, between 01 August 2018 and 31 January 2020. Eligibility was assessed via medical records. Women were approached in antenatal clinics and invited to take part in the research study by clinical and research staff. An information sheet and consent form were provided. Women were enrolled in‐person or online via a study link, up until the day before they gave birth. Hospital medical records were used to monitor when women gave birth. Women who remained eligible for the study after giving birth were contacted via email and/or post in the first 2 weeks postpartum to complete the questionnaire which collected data on women’s general health and social support (‘About You’); their infants (‘About Your Baby’); and perceptions and practices of infant feeding, including the BAB‐Q.

### Measures and materials

Socio‐demographic and maternal health data were collected via self‐report questionnaires. The UK Index of Multiple Deprivation (IMD) (ONS, 2015) was used as an indicator of socio‐economic status. Data on infant feeding (previous experience and initial feeding) were measured on a proportionate scale of infant feeding (Davie, Chilcot, & Bick, 2018). The General Health Questionnaire (GHQ) 12‐item version (Goldberg & Hillier, 1979; Goldberg, 1988) was used to measure general well‐being and distress in the online sample, while the Edinburgh Postnatal Depression Scale (EPDS; Cox, Holden, & Sagovsky, 1987) and Generalised Anxiety Disorder Scale (GAD‐7; Spitzer, Kroenke, Williams, & Lowe, 2006) were used in the cohort sample. Data on infant characteristics were collected via questionnaire in the online sample and via hospital records in the cohort sample.

### Beliefs about breastfeeding questionnaire

The BAB‐Q is a norm‐referenced questionnaire designed by the authors to operationalize the latent constructs of women’s beliefs about breastfeeding, specifically women’s beliefs about the benefits and effort associated with breastfeeding behaviour. Items on the scale were selected and constructed through a literature review of research that used social cognitive theory to explore infant feeding behaviour. Studies that measured beliefs and cognitive‐based (as opposed to affective‐based) attitudes to predict infant feeding behaviour were reviewed. Attitudes are defined in line with theoretical assumptions of the reasoned action approach (Fishbein & Ajzen, 2010) and are understood as evaluative appraisals (ranging from positive to negative) based on underlying salient beliefs. Such beliefs are conceptualized here as behavioural beliefs: subjective acceptance of truth about a particular behaviour formed through observation, information, and inference from socio‐environmental experiences (Fishbein & Ajzen, 2010). Data were collated to identify the most frequently used items for assessing beliefs about breastfeeding (see Table S1). The questionnaire is theorized to function as a cost–benefit utility model where women’s beliefs about the benefits associated with breastfeeding are balanced against women’s beliefs about incurred efforts. Items selected to measure benefit beliefs referenced the most frequently occurring positive attributes of the behaviour (e.g., maternal bonding). Items selected to measure effort beliefs referenced the most common attributes of breastfeeding identified as unfavourable or challenging (e.g., physical exhaustion). Single items were designed to measure eight of the most common beliefs explored in existing literature (see Table S2). Two additional constructs not explicitly used in previous research were also derived from the literature.

Evidence suggests a common reason for breastfeeding cessation is sole maternal responsibility for feeding (Brown & Jordan, 2013; Brown, Rance, & Bennett, 2015; Brown, Raynor, & Lee, 2011; Leff et al., 1994; Marshall et al., 2007; Rempel, 2004). For example, in qualitative interviews women identified one of the main disadvantages of breastfeeding was a constrain on their freedom and autonomy (Murphy, 2000). On‐demand breastfeeding, where infants have unrestricted access to feed, is currently recommended as best practice for the successful initiation and maintenance of exclusive breastfeeding (WHO, 2018a). Feeding infants on‐demand does not necessarily lend to a predictable routine where women can schedule time apart from infants, and this is acknowledged and represented in items currently used (Brown & Jordan, 2013; Brown et al., 2015; Leff et al., 1994; Martens & Young, 1997). This evidence suggested an important construct to consider in women’s beliefs is the concept of women being able to leave infants for some time. This belief is explicitly explored in item 10 of the questionnaire.

Finally, the concept of breastfeeding as an emotionally challenging experience is often neglected (Meneses, 2013) and not explored in current questionnaires. This is acknowledged and explored in the BAB‐Q through item 8 based on items included in the Maternal Breastfeeding Evaluation Scale (Leff et al., 1994) and data from qualitative interviews with breastfeeding women (Brown et al., 2011; Marshall et al., 2007; Sheehan et al., 2013; Shloim et al., 2015).

Items included in the questionnaire used explicit declarative statements about breastfeeding behaviour to ensure clarity and affirmative responses (DeVellis, 1991). In line with the assumptions of norm‐referenced scales (Pett, Lackey, & Sullivan, 2003), the level of measurement is a 5‐point Likert scale of agreeability, giving enough variance to capture both the intensity of agreement or disagreement and the option that respondents may be undecided or indifferent in their beliefs. Items were displayed with no numeric value. Ten items were theorized to sit across two subscales (‘effort’ and ‘benefit’) that sum to create single scores for each scale. Items on each subscale were scored independently using positive integers giving a total score between 5 and 25 for each. Lower scores reflect lower perceived benefits and efforts, and increasing scores represent increased perceived benefit and effort. An indication of the relative importance of these beliefs is calculated by the difference between benefit and effort scores, leaving a benefit–effort differential. This differential score is interpreted as each individuals’ ‘benefit versus effort’ analysis where a positive score indicates perceived benefits as outweighing the effort, and a negative score indicates the efforts as outweighing any benefits. The BAB‐Q was reviewed by a small PPI group (*n* = 4) via email before study commencement. The group were multiparous women with previous personal experience of infant feeding. No amendments the BAB‐Q were recommended prior to study commencement, and feedback provided suggested the questionnaire could be easily understood.

### Ethical considerations

Ethical approval was awarded by institutional‐level Research Ethics Committee (Ref: LRS‐17/18‐5432) to conduct the online study and by a regional Research Ethics Committee (REC) to conduct the cohort study (Ref: 18/LO/0740).

### Statistical analysis

In order to examine the factor structure of the BAB‐Q, exploratory factor analysis (EFA) and confirmatory factor analysis (CFA) were run in MPlus (Version 7.3) on the online and cohort samples, respectively, using weighted least squares with mean and variance adjustment (WLSMV). Before the EFA was conducted, the Kaiser–Meyer–Olkins measure of sampling adequacy, Bartlett’s test of sphericity, and anti‐image correlations were inspected in SPSS. The EFA used the Geomin rotation (a type of oblique rotation) testing 1 and 2 factor solutions. Assessment of goodness of fit for each model was based on standard structural equation modelling criteria (Hu & Bentler, 1999), including comparative fit index (CFI) values >.95, Tucker–Lewis Index (TLI) values >.95, and root mean square error of approximation (RMSEA) values <.08 demonstrating sufficient model fit (Nunnally & Bernstein, 1994; Thompson, 2004). Reliability of each scale/subscale was assessed using Cronbach’s alpha and item‐total correlation coefficients. Construct validity was assessed by comparison of groups and correlations with theoretically related outcomes of breastfeeding experiences and maternal well‐being. Multivariate regression analyses assessed the predictive validity of the questionnaire in predicting infant feeding behaviour and breastfeeding experiences. Descriptive and appropriate inferential statistics were used to explore sample characteristics.

There remains little consensus on the required sample size required to conduct successful factor analysis (Gorsuch, 1983). Recommendations available for item‐to‐subject ratios vary substantially from 1:3 to 1:20, while others give absolute sample size recommendations (Hogarty, Hines, Kromrey, Ferron, & Mumford, 2005; Pett et al.., 2003; Tab achnick & Fidell, 2001). Considering available evidence, a ratio of 20 participants per item was selected (1:20) resulting in a target sample size of at least 220 women for each sample.

## Results

### Sample characteristics

#### Online sample

A total of 452 women accessed the online survey. *N* = 136 women were screened out prior to questionnaire completion because they did not meet eligibility criteria, meaning *N* = 316 women completed the survey. *N* = 38 were excluded from analysis because their infants were premature (<37^+^0 weeks) (*N* = 2), or older than 3 months. A total of *N* = 278 women were included in analyses.

#### Cohort study sample

A total of *N* = 266 women enrolled in the cohort study (total *N* = 422) completed the questionnaire online or via post. *N* = 2 were excluded from analysis because their infants were older than 3 months, meaning *N* = 264 women were included in analyses.

A summary of demographic and clinical characteristics of women and their infants is presented in Table 1. The study samples were distinct in terms of maternal age, ethnic diversity, maternal birth country, socio‐economic status, previous feeding experience, baby age at completion, delivery type, and early feeding practices. Average levels of distress among the samples were low. Among multiparous women (see Table 2), *N* = 15 women in the online sample and *N* = 19 women in the cohort sample reported previous exclusive breastfeeding experience. Most women (online = 71.79%; cohort = 82.31%) initiated exclusive breastfeeding, and only a small proportion initiated exclusive formula feeding (online = 6.96%; cohort = 1.54%). At time of questionnaire completion, the majority of women were still exclusively breastfeeding (see Table 2), although a proportion were mix feeding. Women’s experiences of breastfeeding in the early postnatal period varied considerably across both samples (see Table 2).

**Table 1:** Demographic and clinical characteristics of women and infants

	Online sample		Cohort sample			Group differences
Women	*M* (*SD*)^a^	Range^a^ (%)		*M* (*SD*)^a^	Range^a^ (%)	Statistic (*p*‐value)
Age (*N* = 278|260)	32.56 (4.92)	18–50		35.14 (4.33)	23–49	*t* = −6.43 *p* < .001
Born in UK (*N* = 276|234)	234	84.8%		129	55.1%	χ^2^ = 54.2 *p* < .001

Ethnicity	*N* = 278		*N* = 237		
Asian	3	1.1%	18	7.6%	χ^2^ = 35.0 *p* < .001
Black	4	1.4%	13	5.5%	
Mixed	5	1.8%	10	4.2%	
Other	3	1.1%	12	5.0%	
White	263	94.6%	184	77.6%	

Marital status	*N* = 278		*N* = 238		
Married / civil partnership	204	73.4%	175	73.5%	χ^2^ = 1.38 *p*: .848
Cohabiting with partner	67	24.1%	57	24.0%	
Not cohabiting with partner	2	0.7%	1	0.4%	
Divorced / separated	1	0.4%	0	0%	
Single	4	1.4%	5	2.1%	

Education level	*N* = 278		*N* = 236		
Secondary school	6	2.2%	3	1.3%	χ^2^ = 30.8 *p* < .001
College	51	18.3%	12	5.1%	
University (UG)	109	39.2%	78	33.1%	
University (PG)	112 (40.3)	40.3%	143	60.6%	
Socio‐economic status^b^ (*N* = 278|263)	6.26 (2.64)	1–10	4.80 (2.15)	1–10	*t* = 7.03 *p* < .001
Primiparous (*N* = 277|240)	135	48.7%	134	55.83%	χ^2^ = 2.6 *p* = .107
Distress (GHQ‐12)	11.98 (5.97)	–	–	–	
Depression (EPDS)	–	–	6.95 (4.34)	–	
Anxiety (GAD‐7)	–	–	3.28 (4.45)	–	
Infants					
Girls (*N* = 277|264)	133	47.8%	122	46.2%	χ^2^ = 0.144 *p* = .704
Baby age (days) (*N* = 278|264)	50.7 (25.0)	1–90	22.89 (11.19)	8–72	*t* = 16.32 *p* < .001
Gestational age at birth (*N* = 278|264)	280.59 (8.2)	259–295	279.1 (8.2)	259–296	*t* = 2.00 *p* = .046
Birthweight (kgs) (*N* = 278|264)	3.55 (0.46)	2.40–4.79	3.64 (0.52)	2.21–4.80	*t* = 2.19 *p* = .029

Delivery type	*N* = 277		*N* = 263		
Vaginal (unassisted)	183	66.1%	101	38.4%	χ^2^ = 41.94 *p* < .001
Vaginal (assisted)	33	11.9%	52	19.8%	
Caesarean (planned)	26	9.4%	52	19.8%	
Caesarean (unplanned)	35	12.6%	58	22.1%	

^a^ Figures shown for categories are shown in totals and percentages.

^b^ IMD‐10 = Index of Multiple Deprivation measures relative deprivation across each *output area* in England, Scotland, Wales, and Northern Ireland, and is ranked in percentage deciles from highest deprivation (1) to lowest deprivation (10) using cumulative evidence about income, employment, education, health, crime, and living environments to reflect relative deprivation.

**Table 2:** Summary of infant feeding practices and perceptions

	Online sample	Cohort sample	Group differences
	*N* (%) [Mean (*SD*)]	*N* (%) [Mean (*SD*)]	Statistic (*p*‐value)
Previous feeding experience			
Previous breastfeeding total^a^	*N* = 142	*N* = 103	*t* = −2.74 *p* = .007
	8.37 (5.59)	10.39 (5.82)	
Initial feeding (first 48 hrs)^b^	*N* = 273	*N* = 260	*t* = −2.98
	8.45 (3.14)	9.16 (2.28)	*p* = .003

Current feeding (last 48)	*N* = 278	*N* = 263	
Exclusively formula feeding	35 (12.59)	8 (3.04)	χ^2^ = 18.27 *p *= .001
Mostly formula feeding	12 (4.32)	17 (6.46)	
Equal mixed feeding (50%/50%)	7 (2.52)	6 (2.28)	
Mostly breastfeeding	28 (10.07)	35 (13.31)	
Exclusively breastfeeding	196 (70.50)	197 (74.90)	

Breastfeeding experience	*N* = 278	*N *= 256	
Much more negative than anticipated	48 (17.27)	26 (10.16)	*t* = −1.47 *p* = .140
A little more negative than anticipated	49 (17.63)	39 (15.23)	
As expected	64 (23.02)	74 (28.91)	
A little more positive than anticipated	41 (14.65)	54 (21.09)	
Much more positive than anticipated	76 (27.34)	63 (24.61)	

Beliefs about breastfeeding^c^	*N* = 278	*N* = 264	
Perceived benefit	17.77 (2.62)	17.65 (2.11)	*t* = 0.09 *p* = .930^d^
Perceived effort	13.10 (3.66)	13.02 (3.37)	
Differential score	4.67 (5.19)	4.63 (4.50)	

^a^ Total breastfeeding experience with previous children calculated as average % of breastfeeding in first 6 months of life with previous children.

^b^ Average % breastfeeding in first 48 hrs compared with formula feeding.

^c^ Beliefs about breastfeeding scores following EFA and CFA, using 8‐item, 2‐factor questionnaire.

^d^ *t*‐Test result for differential score.

#### Beliefs about breastfeeding

A summary of responses to the BAB‐Q ise displayed in Table S2. Across both samples, the majority of women strongly believed breastfeeding provided health benefits for babies and develops a close maternal–infant bond. Women also tended to agree (strongly agree or agree) that breastfeeding was rewarding as a mother, saved time and money, but also that breastfeeding was exhausting. Beliefs were more equally distributed across other items in the questionnaire.

## Construct validity

### Exploratory factor analysis

The 10 items taken from the online study were first examined in relation to sample adequacy, which was satisfactory (Kaiser–Meyer–Olkins coefficient = 0.76, Bartlett’s test, χ^2^ = 919.5, df = 45, *p* < .010). Item 5, however, showed a low correlation in the anti‐image matrix (0.63), so it was decided to remove this item. The remaining nine items were subjected to EFA. Inspection of the eigen values computed from MPlus (and subsequent inspection of the scree plot from SPSS) suggested a 2‐factor solution (factor 1 and factor 2 eigenvalues = 4.16 and 1.55, respectively). The 1‐factor EFA model had poor model fit (χ^2^ = 399.5, df = 27, *p* < .010; CFI = .80, TLI = .73, and RMSEA = .22). A 2‐factor model showed better fit (χ^2^ = 99.2, df = 19, *p* < .010; CFI = .96, TLI = .92), albeit the RMSEA was 0.12. Inspection of the rotated factor loadings showed that all values loaded significantly onto a factor, with the exception of item 7 which showed a significant double loading of 0.40. Given this, item 7 was removed and the EFA re‐examined. This shortened 2‐factor model had good fit (χ^2^ = 23.3, df = 13, *p* < .040; CFI = .99, TLI = .99, and RMSEA = .05), with all factors loading significant (see Table 3). Factor 1 (benefit beliefs) and factor 2 (effort beliefs) accounted for 47 and 19.4% of the explained variance, respectively. The two factors correlated moderately (*r* = −.40).

**Table 3:** Factor loadings from the EFA (Geomin rotation) using online sample data

Item	Factor 1 Benefit beliefs	Factor 2 Effort beliefs
1. Breastfeeding provides many health benefits for babies	0.89*	
2. Breastfeeding develops a close bond between mother and baby	0.93*	
3. The lifestyle changes mothers make for breastfeeding are inhibiting		0.54*
4. Breastfeeding is rewarding for mothers	0.70*	
6. Breastfeeding is exhausting		0.98*
8. Breastfeeding is emotionally draining		0.85*
9. Breastfeeding saves time and money	0.45*	
10. Breastfeeding means mothers can’t leave their babies		0.40*

* *p*< .050.

### Confirmatory factor analysis

**Figure 1:**
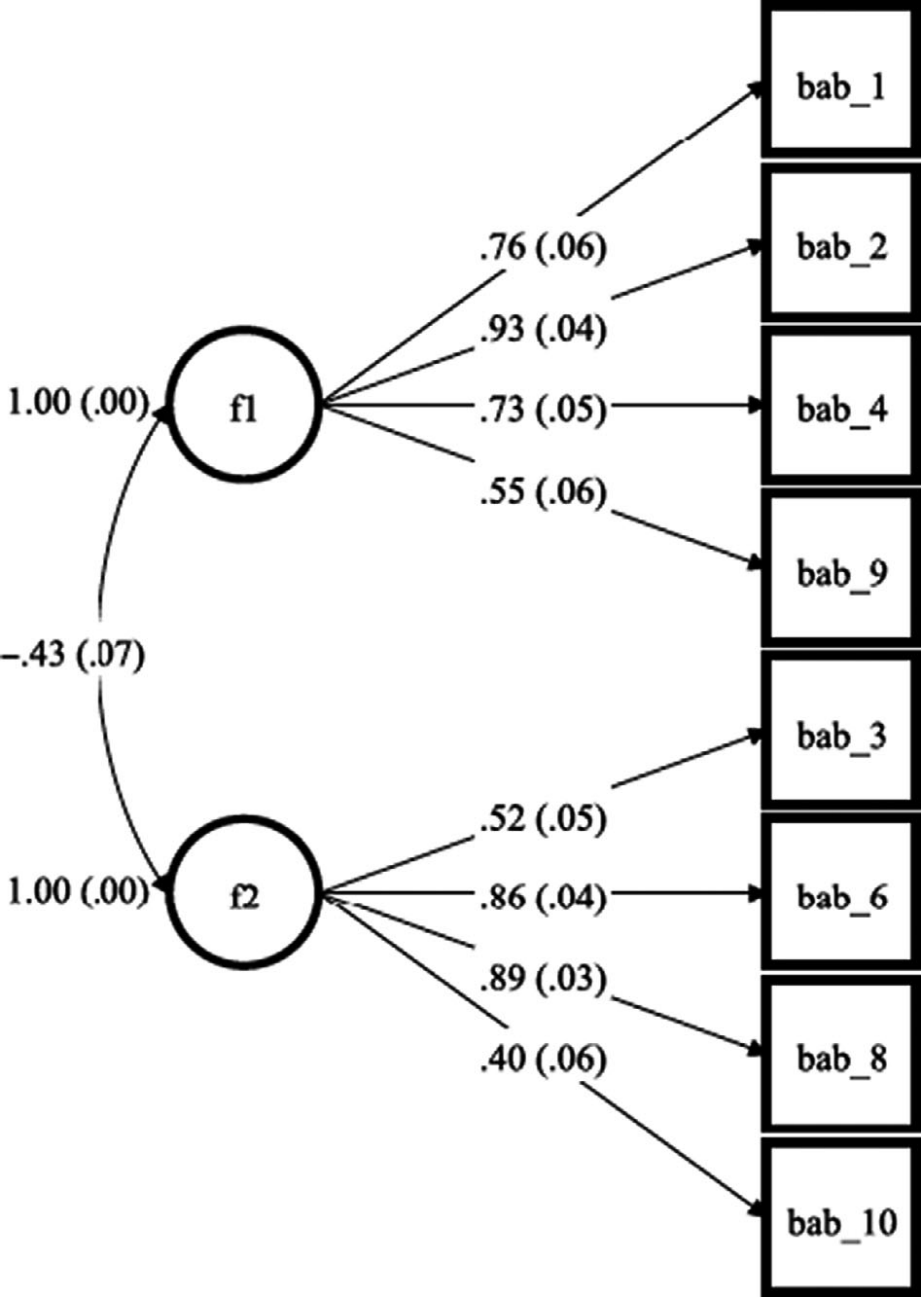
CFA of The BAB‐Q using data from the cohort sample.

CFA was conducted on the 8‐item, 2‐factor specification identified above. The 2‐factor model showed good model fit (χ^2^ = 49.6 df = 19, *p* < .010; CFI = .97, TLI = .96, and RMSEA = .078); see Figure 1 for standardized factor loadings. The two latent factors correlated negatively (*r* = −.43).

### Comparison of groups

Using the 2‐factor, 8‐item model, it was theoretically hypothesized that women with higher differential scores would have more positive breastfeeding experiences and better well‐being, and women with lower differential scores would have more negative breastfeeding experiences and worse well‐being.

In the online sample, a one‐way analysis of variance (ANOVA) with Tukey’s *post‐hoc* comparison found differential scores differed significantly depending on breastfeeding experience, *F*(4,278) = 29.21, *p* < .001. Average differential scores were significantly lower among women with ‘much more negative’ experiences (*M* = −0.42, *SD* = 4.53) than women who experienced breastfeeding ‘as expected’ (*M* = 4.83, *SD* = 5.24; *p* < .001, 95% CI [2.95, 7.54]) and significantly higher in women who experienced breastfeeding as ‘much more positive’ (*M* = 8.09, *SD* = 3.60; *p* < .001, 95% CI [1.23, 5.30]). Lower differential scores were signifiantly correlated with increased maternal distress (*r* = −.45, *p* < .001). Analyses found comparable results in the cohort sample, *F*(4,256) = 23.23, *p* < .001. Average differential scores were significantly lower among women with ‘much more negative’ experiences (*M* = −0.96, *SD* = 4.42) than women who experienced breastfeeding ‘as expected’ (*M* = 4.86, *SD* = 3.74; *p* < .001, 95% CI [3.34, 8.27]) and significantly higher in women who experienced breastfeeding as ‘much more positive’ (*M* = 7.51, *SD* = 4.18; *p* = .001, 95% CI [0.80, 4.48]). Lower differential scores were significantly correlated with increased maternal depression (*r* = −.38, *p* < .001) and anxiety (*r* = −.32, *p* < .001).

### Reliability

For the modified 8‐item BAB‐Q, Cronbach’s alpha coefficient of .73 was obtained in the online sample and .77 in the cohort sample, which exceeded the recommended .70 for new instruments (Nunnally & Bernstein, 1994) and indicated good internal consistency. Each subscale showed good reliability with Cronbach’s alpha values of .70 (benefits) and .72 (effort) in the online sample, and .78 (benefits) and .75 (efforts) in the cohort sample. Item‐total correlations ranged between .50 and .68 in the online sample, and .55 and .70 in the cohort sample, which were all within the acceptable range (Nunnally & Bernstein, 1994).

### Predictive validity

#### Predicting breastfeeding behaviour

##### Online sample

An adjusted multinomial regression model predicted infant feeding practices based on beliefs about breastfeeding, *F*(36,141) = 90.29, *p* < .001; pseudo *R* ^2^ = 37.96. An increase in women’s benefit beliefs significantly increased the likelihood of exclusive breastfeeding with a relative risk (RR) = 1.75 (*p* < .001), compared with exclusive formula feeding. However, women’s benefit beliefs did not significantly predict the likelihood of women predominantly formula feeding (RR = 1.18, *p* = .537), mix feeding (RR = 1.12, *p* = .534), or predominantly breastfeeding (RR = 1.34, *p* = .101). Women’s effort beliefs did not significantly predict the likelihood of predominant formula feeding (RR = 1.03, *p* = .907), mix feeding (RR = 1.12, *p* = .534), predominant breastfeeding (RR = 1.34, *p* = .101), or exclusive breastfeeding (RR = 0.88, *p *= .329) (see Table 4). Regression models using differential scores (see Table S3) showed comparable results and indicated a one‐point increase in differential score was associated with a significantly increased likelihood (RR = 1.38, *p* < .001) of women exclusively breastfeeding, rather than exclusively formula feeding.

**Table 4:** Online sample‐adjusted multinomial regression analyses of infant feeding practices and beliefs about breastfeeding

	RR	*SE*	*p*	95% CI
Predominant formula feeding				
Previous feeding	1.07	0.23	.744	0.71, 1.62
Maternal distress	0.91	0.15	.570	0.65, 1.26
BAB benefit	1.18	0.31	.537	0.70, 1.96
BAB effort	1.03	0.26	.907	0.63, 1.68
_cons	7.70^e‐16^	1.33^e‐14^	.044	0.00, 0.42
Mix feeding				
Previous feeding	1.26	0.17	.147	0.93, 1.61
Maternal distress	1.06	0.08	.427	0.91, 1.24
BAB benefit	1.40	0.33	.161	0.88, 2.23
BAB effort	1.12	0.21	.534	0.78, 1.61
_cons	2.18^e‐09^	2.29^e‐08^	.057	0.00, 1.84
Predominant breastfeeding				
Previous feeding	1.48	0.19	.002*	1.15, 1.91
Maternal distress	0.96	0.08	.616	0.82, 1.13
BAB benefit	1.16	0.19	.367	0.84, 1.61
BAB effort	1.34	0.25	.101	0.94, 1.93
_cons	7.49^e‐08^	6.62^e‐07^	.063	0.00, 2.50
Exclusive breastfeeding				
Previous feeding	1.48	0.16	<.001*	1.20, 1.82
Maternal distress	1.00	0.06	.998	0.89, 1.13
BAB benefit	1.75	0.28	<.001*	1.29, 2.39
BAB effort	0.88	0.12	.329	0.68, 1.14
_cons	5.52^e‐10^	4.02^e‐09^	.003	0.00, 0.00

CI = confidence interval; *p* = *p*‐value; RR = risk ratio; *SE* = standard error.

Exclusive formula feeding as reference category. Underlying assumptions of independence of observations (Durbin–Watson [10, 141] = 1.19) and multicollinearity (mean VIF = 1.14) were met, and no severe outliers were observed in the data. Regression model adjusted for maternal age, socio‐economic status, baby age, baby birthweight, and delivery type. Delivery method collapsed into two groups (vaginal vs caesarean) due to low cell frequencies.

* *p*‐value significant at α = .05.

##### Cohort sample

Infant feeding data were standardized using *z*‐scores to correct for negative skew and kurtosis (χ^2^ = 134.99, df = 109, *p* = .046). Adjusted linear regression analyses, *F*(17,79) = 2.00, *p* = .020; adjusted *R* ^2^ = 15.06) showed women’s beliefs about breastfeeding did not significantly predict infant feeding practices. Benefit beliefs (β = .05, *p* = .372) or efforts beliefs (β = .004, *p* = .927) were not significantly associated with feeding practice (see Table 5). Regression models using differential scores (see Table S4) showed no significant association with infant feeding practices (β = .02, *p* = .600).

**Table 5:** Cohort sample‐adjusted linear regression analyses of infant feeding practices^a^ and beliefs about breastfeeding

	β‐coefficient	*SE*	*p*	95% CI
Ethnicity^b^				
Asian	0.02	0.39	.960	‒0.76, 0.80
Black	0.11	0.53	.834	‒0.94, 1.16
Mixed	‒0.29	0.47	.537	‒1.21, 0.64
Other	0.41	0.55	.461	‒0.69, 1.50
Marital status^c^				
Married / civil partnership	1.32	0.77	.092	‒0.22, 2.86
Cohabiting with partner	1.34	0.79	.093	‒0.23, 2.92
Partnered, not cohabiting	1.08	1.36	.433	‒1.64, 3.79
Maternal age	0.01	0.03	.766	‒0.05, 0.07
Socio‐economic status	0.02	0.06	.740	‒0.09, 0.13
Previous feeding	0.07	0.02	.001*	0.03, 0.12
Depression	0.01	0.04	.754	‒0.07, 0.10
Anxiety	‒0.07	0.06	.224	‒0.18, 0.04
Delivery^d^				
Vaginal assisted	0.56	0.35	.117	‒0.14, 1.25
Caesarean planned	‒0.01	0.28	.979	‒0.57, 0.56
Caesarean unplanned / emergency	0.21	0.39	.584	‒0.56, 0.98
BAB benefit	0.05	0.05	.372	‒0.06, 0.15
BAB effort	0.004	0.04	.927	‒0.08, 0.08
_cons	‒3.31	1.68	.052	‒6.65, 0.03

CI = confidence interval; *p* = *p*‐value; *SE* = standard error.

^a^ Standardized infant feeding scores (*z*‐scores) used.

^b^ ‘White’ as reference base category.

^c^ ‘Single’ as reference category.

^d^ Category ‘Vaginal unassisted’ as reference base category. Assumptions of independence of observations (Durbin–Watson [18, 97] = 1.01) and multicollinearity (mean VIF = 2.72) were met, and no outliers were observed in the data.

* *p*‐value significant at α = .05.

#### Predicting breastfeeding experiences

##### Online sample

A multinomial regression model adjusted for maternal health and delivery method predicted breastfeeding experiences based on beliefs about breastfeeding, χ^2^(24,277) = 128.31, *p* < .001, pseudo *R* ^2^ = 14.63, (see Table 6). An increase in women’s benefit beliefs significantly reduced the likelihood that women experienced breastfeeding as ‘much more negative’ (RR = 0.86, *p* = .039) than expected, whereas an increase in effort beliefs was associated with a significantly increased likelihood that women experienced breastfeeding as ‘much more negative’ (RR = 1.22, *p* = .006) than they expected. In regression models using differential scores (see Table S5), a one‐point increase in differential score was associated with a significantly reduced risk (RR = 0.83, *p* < .001) of women experiencing breastfeeding as ‘much more negative’ than they expected. For positive breastfeeding experiences, an increase in women’s benefit beliefs significantly increased the likelihood that women experienced breastfeeding as ‘much more positive’ (RR = 1.30, *p* = .009). An increase in women’s effort beliefs significantly reduced the likelihood women experienced breastfeeding as ‘much more positive’ (RR = 0.87, *p* = .020). A one‐point increase in differential score (see Table S5) was associated with a significantly increased risk (RR = 1.20, *p* < .001) of women experiencing breastfeeding as ‘much more positive’ than they expected. Women’s beliefs did not significantly predict experiences with less emotional valence (i.e., a little more positive/negative) (see Table 6).

**Table 6:** Adjusted multinomial regression analyses of breastfeeding experiences and beliefs about breastfeeding among online and cohort samples

Experience by group		RR	*SE*	*p*	95% CI
Much more negative					
Online	BAB benefit	0.86	0.06	.039*	0.74, 0.99
	BAB effort	1.22	0.09	.006*	1.06, 1.40
	_cons	0.07	0.13	.136	0.002, 2.29
Cohort	BAB benefit	0.72	0.09	.006*	0.57, 0.91
	BAB effort	1.40	0.16	.002*	1.13, 1.75
	_cons	0.14	0.40	.489	0.001, 36.38
A little more negative					
Online	BAB benefit	1.07	0.09	.417	0.91, 1.27
	BAB effort	1.08	0.07	.257	0.95, 1.23
	_cons	0.03	0.06	.076	0.001, 1.43
Cohort	BAB benefit	0.96	0.10	.704	0.78, 1.19
	BAB effort	1.10	0.08	.194	0.95, 1.27
	_cons	0.20	0.47	.491	0.002, 19.86
A little more positive					
Online	BAB benefit	1.03	0.09	.724	0.87, 1.21
	BAB effort	0.92	0.06	.189	0.80, 1.04
	_cons	0.49	0.90	.697	0.01, 18.41
Cohort	BAB benefit	0.90	0.09	.287	0.74, 1.09
	BAB effort	0.95	0.06	.379	0.83, 1.07
	_cons	7.05	14.98	.357	0.11, 452.24
Much more positive					
Online	BAB benefit	1.31	0.14	.009*	1.07, 1.61
	BAB effort	0.87	0.05	.020*	0.77, 0.98
	_cons	0.06	0.14	.221	0.00, 5.27
Cohort	BAB benefit	1.25	0.15	.060	0.99, 1.59
	BAB effort	0.86	0.05	.019*	0.76, 0.96
	_cons	0.07	0.17	.283	0.001, 9.12

Base outcome = ‘My experience with breastfeeding so far has been As I Expected’; CI = confidence interval; *p* = *p*‐value; RR = relative risk; *SE* = standard error.

Analyses were adjusted for delivery method, maternal well‐being (online sample), and maternal depression and maternal anxiety (cohort sample). Online Sample underlying assumptions of independence of observations (Durbin–Watson [7, 277] = 1.89) and multicollinearity (mean VIF = 1.19) were met. Cohort sample underlying assumptions of independence of observations (Durbin–Watson [8, 250] = 1.79) and multicollinearity (mean VIF = 1.61) were met.

* *p*‐value significant at α = .05.

#### Cohort sample

A multinomial regression model adjusted for maternal health and delivery method predicted breastfeeding experiences based on beliefs about breastfeeding, χ^2^(28,250) = 115.98, *p* < .001, pseudo *R* ^2^ = 14.92. Women’s increasing effort beliefs significantly increased the likelihood that women experienced breastfeeding as ‘much more negative’ (RR = 1.40, *p* = .002) than expected. Conversely, increased benefit beliefs significantly decreased the likelihood of women experiencing breastfeeding as ‘much more negative’ (RR = 0.72, *p* = .006) (see Table 6). Adjusted regression model (see Table S5) showed a one‐point increase in differential score was associated with a significantly reduced risk (RR = 0.72, *p* < .001) of women experiencing breastfeeding as ‘much more negative’ than they expected. For positive breastfeeding experiences, a decrease in effort beliefs was associated with a significantly increased likelihood that women experienced breastfeeding as ‘much more positive’ (RR = 0.86, *p* = .019), than they expected. However, an increase in benefit beliefs did not significantly increase the likelihood of a ‘much more positive’ experience (RR = 1.25, *p* = .060). A one‐point increase in differential score (see Table S5) was associated with a significantly increased likelihood (RR = 1.18, *p* = .002) of women experiencing breastfeeding as ‘much more positive’ than they expected. Women’s beliefs about breastfeeding did not significantly predict feeding experiences with less emotional valence (i.e., a little more positive/negative) (see Table 6).

## Discussion

This paper proposed the Beliefs About Breastfeeding Questionnaire for use in research, with potential application to breastfeeding support interventions. A psychometric evaluation was performed assessing the reliability, construct validity, and predictive validity of the measure.

An EFA on the proposed 10‐item questionnaire found a two‐factor model showed good fit, with the removal of two items. One removed item in the questionnaire aimed to measure beliefs that ‘Mothers are responsible for all the feeds with breastfeeding’. This item is used throughout current research, but depending on how breastfeeding is valued in women’s socio‐cultural environment, the item may be appraised as a positive or negative attribute of breastfeeding, which may explain the low correlation in the matrix. Where breastfeeding is appraised as empowering for women (Groleau, Pizarro, Molino, Gray‐Donald, & Semenic, 2017), assimilated with an ideology of motherhood (Marshall et al., 2007; Murphy, 2000), and supported as a normal practice, maternal responsibility may be appraised as inherently positive. However, in many Western socio‐cultural environments, as in this study, shared parental responsibility of childrearing (including infant feeding) is expected (Emmott & Mace, 2015) and breastfeeding is not the social norm (McAndrew et al., 2012; Smyth, 2012), so sole maternal responsibility for infant feeding may be perceived as negative attribute of breastfeeding. The item may also have been ambiguous as women can express breastmilk to share feeding responsibilities with partners, family, or friends. The item *‘*Breastfeeding allows you to go places and do things outside the home easily’ was also removed. Quantitative and qualitative findings show that even though breastfeeding is considered convenient (Brown & Lee, 2011) many women experience apprehension, embarrassment, and judgement when they breastfeed in public (Thomson, Ebisch‐Burton, & Flacking, 2015). By association, this item may therefore have tapped into beliefs about breastfeeding in public alongside convenience beliefs, which may explain the dual loading on both effort and benefit factors. These two removed items have been used throughout infant feeding research to date but were not reliable or valid within the BAB‐Q. Overall, EFA provided a parsimonious set of factors that concisely summarized the underlying structure of beliefs about breastfeeding on an effort–benefit framework.

Confirmatory factor analysis on the shortened 8‐item BAB‐Q confirmed the construct validity of the questionnaire and showed factor 1 (benefit beliefs) and factor 2 (effort beliefs) were negatively correlated. The factors showed good reliability in both samples, and item‐total correlations for benefit and effort subscales were consistent with theory. This provides support for the assumption that women can hold beliefs that appraise breastfeeding as both beneficial and effortful at the same time, which contradicts assumptions of the IIFAS (Mora et al., 1999). The construct validity of the questionnaire also suggests women represent breastfeeding behaviour on a cost–benefit framework as observed in core components of some social‐cognition models (Conner & Norman, 2005) rather than a result of sequential reasoned action. Correlations with theoretically related outcomes further supported the questionnaire construct validity and found maternal depression, anxiety, and distress were significantly correlated with BAB differential scores where effort beliefs trended to outweigh benefit beliefs. In this cross‐sectional study, the causality of the association remains unknown, but these associations are consistent with previous research which suggest women with depressive symptoms have shorter breastfeeding durations and more difficult experiences (Brown et al., 2015; Watkins, Meltzer‐Brody, Zolnoun, & Stuebe, 2011). Comparison of groups provided additional support for construct validity of the questionnaire, which found different benefit and effort beliefs among women who had breastfeeding experiences that differed drastically from what they expected. These associations were confirmed by findings that assessed the predictive validity of the questionnaire.

The BAB‐Q significantly predicted women’s breastfeeding experiences. Specifically, women with increased benefit beliefs were more likely to have ‘much more positive’ experience and less likely to have a negative breastfeeding experience. Conversely, women with increased effort beliefs were more likely to have a ‘much more negative’ breastfeeding experience and less likely to have positive experiences. All associations reached statistical significance with the exception of benefit beliefs predicting much more positive experiences in the cohort sample. Women’s beliefs about breastfeeding also did not significantly predict experiences with less emotional valence (i.e., a little more positive/negative). The cross‐sectional nature of the study means the direction of associations observed is unclear, and women’s difficulties with breastfeeding (such as nipple pain or low milk supply) were not accounted for, so any confounded effects remain unknown. Existing evidence suggests that when breastfeeding is promoted unrealistically and does not account for the realities of motherhood or the challenges new mothers can expect, women feel unprepared and can experience additional difficulties with breastfeeding (Fox, McMullen, & Newburn, 2015; Hegney, Fallon, & O’Brein, 2008; Leurer & Misskey, 2015; Trickey & Newburn, 2014). It is therefore plausible that women’s beliefs about breastfeeding precede their experiences. If women believe breastfeeding to be a solely beneficial experience, and do not anticipate breastfeeding to be difficult or effortful, their experiences may be more negative than they had expected when they encounter difficulties or the realities of infant feeding (Hengney et al., 2008). Given the reliability and validity of the questionnaire currently presented, the BAB‐Q may provide guidance for healthcare providers to direct additional care and support to women who have very negatively or positively skewed beliefs to ensure they are informed of the benefits, and well‐prepared for the potential challenges of breastfeeding, respectively.

Despite the observed reliability and construct validity, the BAB‐Q did not significantly or reliably predict women’s infant feeding practices. These results are not consistent with existing evidence that show attitudes to breastfeeding predict infant feeding practices across the postpartum period (McMillan et al., 2008; Scott et al., 2006; Zhu et al., 2017). Existing evidence highlights women will continue to breastfeed even when it leads to physical or psychological pain and distress, in order to achieve their goals (Hegney et al., 2008; McAndrew et al., 2012). Breastfeeding is valuable to women, and it is often considered an idealism of motherhood (Marshall et al., 2007; Murphy, 2000) where the needs of the infant are prioritized over and above those of the mother (Shloim et al., 2015). Therefore, even when women experience increased efforts of breastfeeding, women may embrace this aspect of motherhood and persevere to continue breastfeeding. The average differential scores were positive and suggest women in this study believed breastfeeding to be more beneficial than effortful. It is therefore possible that there is a threshold where efforts have to far outweigh the benefits, over and above the average differential scores observed here, before changes in feeding practice are observed. This is supported by the observation that the majority of women (>80%) in the study were predominantly breastfeeding their infants. The bias in infant feeding practices and average BAB differential scores may, in part, explain the lack of predictive validity of the BAB‐Q at predicting breastfeeding and formula‐feeding behaviour. Examining the predictive validity of the questionnaire among women with more diverse feeding practices and beliefs is necessary. A longitudinal assessment of the predictive validity of the 8‐item questionnaire is also needed to examine whether antenatal beliefs about breastfeeding predict postnatal experiences and behaviour and assess the extent to which women’s beliefs and experiences change over the antenatal and postnatal period.

### Strengths and limitations

Strengths of this study include a large, adequate sample size for conducting both exploratory and confirmatory factor analyses. The psychometrics of the questionnaire were tested in one population, and the solution was confirmed in a separate cohort of postnatal women with distinct demographic characteristics. These results provide support for the use of the questionnaire across demographically diverse samples. The questionnaire was reviewed by an expert PPI group of postnatal women (*N* = 4) prior to use in the online and cohort studies. However, this does not constitute sufficient face validity testing. Additional limitations include the study collected data from two cross‐sectional samples, meaning the directionality of associations cannot be inferred. The online sample relied on maternal self‐report for all questionnaires, and across the whole study, infant feeding practices were self‐reported according to WHO (2003) recall method. As the title of the questionnaire includes ‘breastfeeding’, women may have answered with traits of demand characteristics and over‐reported breastfeeding behaviour. In addition, data on women’s intentions for infant feeding were not collected or controlled for. Finally, construct validity could not be evaluated by correlating the BAB‐Q with other measures of breastfeeding beliefs because questionnaires commonly used have not been validated, do not list their items, or use a limited items without theoretical foundations.

### Conclusion

Breastfeeding is a health behaviour with bio‐psycho‐social origins that provides an early‐life intervention for the health of women and infants across the lifespan. To target women who are likely to stop breastfeeding, modifiable factors associated with behaviour, such as beliefs, need to be identified to guide the development and implementation of individual‐level support interventions. With a lack of psychometrically validated instruments available to measure women’s beliefs about breastfeeding, the BAB‐Q may provide a suitable alternative to outdated and theoretically uninformed items currently used. Psychometric evaluation of the questionnaire in two separate samples of postnatal women suggested it is a reliable measure with good construct validity that consistently predicts women’s breastfeeding experiences that deviate drastically from what they expected. Overall, the utility of the BAB‐Q at predicting breastfeeding behaviour remains unclear and unsupported by empirical evidence. To further assess the predictive utility of the questionnaire, longitudinal studies in populations with diverse beliefs and infant feeding practices are required.

## Conflicts of interest

All authors declare no conflict of interest.

## Author contributions

Philippa Davie, MSc (Conceptualization; Data curation; Formal analysis; Writing – original draft) Debra Bick (Conceptualization; Methodology; Supervision; Writing – review & editing) Joseph Chilcot (Conceptualization; Formal analysis; Supervision; Writing – review & editing).

## Supporting information


**Table S1**. Example items used in existing literature to explore beliefs and attitudes about breastfeeding.
**Table S2**. Responses to the Beliefs About Breastfeeding scale.
**Table S3**. Online sample adjusted multinomial regression analyses of infant feeding practices and beliefs about breastfeeding using BAB differential scores.
**Table S4**. Cohort sample adjusted linear regression analyses of infant feeding practices^†^ and beliefs about breastfeeding using BAB differential scores.
**Table S5**. Adjusted Multinomial regression analyses of breastfeeding experiences and beliefs about breastfeeding among online and cohort samples using BAB differential scores.

## Data Availability

The data that support the findings of this study are available on request from the corresponding author. The data are not publicly available due to privacy or ethical restrictions.
