# Supplementary material for: The Beliefs About Breastfeeding Questionnaire (BAB‐Q): A psychometric validation study
Source: Br J Health Psychol. 2020 Dec 19;26(2):482–504. doi: 10.1111/bjhp.12498 (PMC8247407; doi:10.1111/bjhp.12498)
Supplement: Supplementary file 1 — Table S1. Example items used in existing literature to explore beliefs and attitudes about breastfeeding. Table S2. Responses to the Beliefs About Breastfeeding scale. Table S3. Online sample adjusted multinomial regression analyses of infant feeding practices and beliefs about breastfeeding using BAB differential scores. Table S4. Cohort sample adjusted linear regression analyses of infant feeding practices† and beliefs about breastfeeding using BAB differential scores. Table S5. Adjusted Multinomial regression analyses of breastfeeding experiences and beliefs about breastfeeding among online and cohort samples using BAB differential scores. [file BJHP-26-482-s001.docx]

| Online Supplement Table 1 *Example Items Used in Existing Literature to Explore Beliefs and Attitudes About Breastfeeding* | |
| --- | --- |
| Construct | Example Items |
| Health/ Nutritional Benefits of breastfeeding (Item 1) | Breastfeeding is best for baby’s health^3^ Breastfeeding helps protect the baby from getting sick and having allergies^5,6^ Breastfeeding is the healthiest feeding for a baby^5,6^  Babies fed breastmilk are healthier than babies who are fed formula^9^ |
| Breastfeeding promotes bonding with baby (Item 2) | Breastfeeding will help me feel close to my baby^5,6^ Breastfeeding increases mother-infant bonding^9^  I felt extremely close to my baby when I breastfed^7^ Breastfeeding would make you and your baby develop close feelings^11^ |
| Changes to lifestyle associated with accommodating breastfeeding (Item 3) | I wanted a more predictable routine^1,2,3^ You have more of a routine if you formula feed^3^ I could easily fit my baby’s breastfeeding with other activities^7^ Breastfeeding limits a mother’s social life^13^  Breastfeeding means I can’t go back to work or school^5,6^ A mother who occasionally drinks alcohol should not breastfeed her baby^9^ Breastfeeding means I have to eat differently^5,6^ |
| Rewarding as a mother (Item 4) | Breastfeeding would make you feel good about yourself^11^  The fact that I could produce the food to feed my own baby was very satisfying^7^  Breastfeeding was a very nurturing maternal experience^7^  Breastfeeding make me feel more confident as a mother^7^ |
| Maternal responsibility for breastfeeding (Item 5) | Breastfeeding means no one else can feed the baby^5,6^  If you formula feed other people can feed the baby^3^ Breastfeeding is too much responsibility^3^ I didn’t like being responsible for all the feeds^1,2,3^ It was a burden being my baby’s main source of food^7^ Being baby’s source of food was a burden^7^  Felt too tied down^7^ |
| Physical easy/difficulty of breastfeeding compared to formula feeding (Item 6) | Breastfeeding was physically demanding^7^ Breastfeeding is too much hassle^3^ Breastfeeding is difficult^3^ I was exhausted^1,2,3^ With breastfeeding I felt too tied down all the time^7^ |
| Mobility of breastfeeding outside the home (Item 7) | I was stuck in the house breastfeeding^1,2,3^ Breastfeeding in a public place is acceptable^8^ Formula feeding is the better choice if a mother chooses to work outside the house^9^ Breastfeeding would allow you to go places and do thinks outside the home easily^11^ |
| Financial cost and time burden associated with formula feeding (Item 9) | Breastfeeding is cheaper than using formula^5,8^  Breastfeeding is more convenient than formula feeding^8,9^ Breastfeeding takes too much time^3^ Breastfeeding would not cost very much money^11^ |
| ^1^Brown & Jordan (2013); ^2^Brown, Rance & Bennett (2015); ^3^Brown, Raynor & Lee (2011); ^4^DeJager et al. (2015); ^5^Kloeblen, Thompson & Miner (1998); ^6^Humphreys, Thompson & Miner (1998); ^7^Leff, Jefferis & Gagne (1994); ^8^Lou et al. (2014); ^9^Mora et al., (1999); ^10^O’Brein, Buikstra & Hegney (2008); ^11^Semenic, Loiselle & Gottlieb (2008);^12^Shepheard, Walbey & Lovell (2017); ^13^Swanson & Power (2005). | |

| Online Supplement Table 2 *Responses to the Beliefs About Breastfeeding scale* | | | | | | | | | | |  |
| --- | --- | --- | --- | --- | --- | --- | --- | --- | --- | --- | --- |
| BAB Questionnaire Items | Group | Strongly Disagree | | Disagree | | Neutral | | Agree | Strongly Agree | Total | |
| 1. Breastfeeding provides many health benefits for babies | 1 | 2 | 2 | | 6 | | 37 | | 231 | 278 | |
|  | 2 | 0 | 0 | | 6 | | 32 | | 226 | 264 | |
| 2. Breastfeeding develops a close bond between mother and baby | 1 | 6 | 1 | | 26 | | 56 | | 189 | 278 | |
|  | 2 | 0 | 3 | | 10 | | 56 | | 195 | 264 | |
| 3. The lifestyle changes mothers make for breastfeeding are inhibiting | 1 | 35 | 57 | | 52 | | 96 | | 38 | 278 | |
|  | 2 | 18 | 49 | | 63 | | 93 | | 40 | 263 | |
| 4. Breastfeeding is rewarding for mothers | 1 | 4 | 14 | | 34 | | 81 | | 145 | 278 | |
|  | 2 | 5 | 6 | | 39 | | 108 | | 106 | 264 | |
| 5. Mothers are responsible for all the feeds with breastfeeding | 1 | 10 | 60 | | 36 | | 74 | | 98 | 278 | |
|  | 2 | 7 | 70 | | 51 | | 80 | | 56 | 264 | |
| 6. Breastfeeding is exhausting | 1 | 7 | 37 | | 49 | | 104 | | 81 | 278 | |
|  | 2 | 9 | 30 | | 54 | | 103 | | 68 | 264 | |
| 7. Breastfeeding allows you to go places and do things outside the home easily | 1 | 21 | 41 | | 41 | | 94 | | 81 | 278 | |
|  | 2 | 9 | 48 | | 71 | | 83 | | 52 | 263 | |
| 8. Breastfeeding is emotionally draining | 1 | 37 | 60 | | 53 | | 75 | | 53 | 278 | |
|  | 2 | 31 | 71 | | 64 | | 72 | | 25 | 263 | |
| 9. Breastfeeding saves time and money | 1 | 6 | 12 | | 41 | | 73 | | 146 | 278 | |
|  | 2 | 2 | 19 | | 57 | | 88 | | 98 | 264 | |
| 10. Breastfeeding means mothers can’t leave their babies | 1 | 21 | 100 | | 44 | | 84 | | 29 | 278 | |
|  | 2 | 21 | 77 | | 61 | | 78 | | 26 | 263 | |
| 1: Online Sample 2: Cohort Sample; Effort items: 3, 5, 6, 8, 10; Benefit items: 1, 2, 4, 7, 9. | | | | | | | | | | |  |

| Online Supplement Table 3 *Online Sample Adjusted Multinomial Regression Analyses of Infant Feeding Practices and Beliefs About Breastfeeding Using BAB Differential Scores* | | | | |
| --- | --- | --- | --- | --- |
|  | RR | SE | p | 95% CI |
| Predominant Formula Feeding |  |  |  |  |
| Age | 1.76 | 0.58 | .084 | 0.93, 3.35 |
| Socioeconomic status | 1.31 | 0.56 | .537 | 0.56, 3.04 |
| Baby age | 0.97 | 0.03 | .282 | 0.91, 1.03 |
| Baby birthweight | 1.00 | 0.00 | .149 | 1.00, 1.01 |
| Previous feeding | 1.06 | 0.24 | .804 | 0.71, 1.64 |
| Maternal well-being | 0.92 | 0.16 | .628 | 0.66, 1.29 |
| Delivery**^†^** - Caesarean | 0.00^‡^ | 0.00^‡^ | .987 | 0.00, 0.00^‡^ |
| BAB Differential | 1.07 | 0.22 | .740 | 0.72, 1.59 |
| Mix-feeding |  |  |  |  |
| Age | 1.17 | 0.16 | .264 | 0.89, 1.54 |
| Socioeconomic status | 1.07 | 0.22 | .743 | 0.71, 1.61 |
| Baby age | 0.98 | 0.02 | .359 | 0.94, 1.02 |
| Baby birthweight | 1.00 | 0.001 | .458 | 1.00, 1.00 |
| Previous feeding | 1.17 | 0.15 | .245 | 0.90, 1.51 |
| Maternal well-being | 1.07 | 0.08 | .350 | 0.93, 1.23 |
| Delivery - Caesarean | 0.77 | 0.90 | .852 | 0.08, 7.51 |
| BAB Differential | 1.09 | 0.14 | .512 | 0.85, 1.40 |
| Predominant Breastfeeding |  |  |  |  |
| Age | 1.08 | 0.14 | .521 | 0.85, 1.39 |
| Socioeconomic status | 0.83 | 0.16 | .350 | 0.56, 1.26 |
| Baby age | 0.96 | 0.02 | .082 | 0.92, 1.00 |
| Baby birthweight | 1.00 | 0.001 | .160 | 1.00, 1.00 |
| Previous feeding | 1.41 | 0.17 | .004* | 1.12, 1.78 |
| Maternal well-being | 0.99 | 0.72 | .934 | 0.86, 1.15 |
| Delivery - Caesarean | 0.44 | 0.50 | .468 | 0.05, 4.02 |
| BAB Differential | 0.95 | 0.11 | .654 | 0.76, 1.19 |
| Exclusive Breastfeeding |  |  |  |  |
| Age | 1.24 | 0.12 | .026* | 1.03, 1.49 |
| Socioeconomic status | 1.03 | 0.14 | .856 | 0.78, 1.35 |
| Baby age | 0.97 | 0.02 | .101 | 0.94, 1.01 |
| Baby birthweight | 1.00 | 0.001 | .112 | 1.00, 1.00 |
| Previous feeding | 1.41 | 0.13 | <.001* | 1.17, 1.70 |
| Maternal well-being | 1.01 | 0.06 | .852 | 0.90, 1.14 |
| Delivery - Caesarean | 0.28 | 0.24 | .137 | 0.05, 1.49 |
| BAB Differential | 1.38 | 0.14 | .001* | 1.14, 1.68 |
| Exclusive Formula Feeding as reference category. RR = Risk Ratio; SE = Standard Error; p = p-value significant at α= .05; CI = Confidence Interval. **^†^**Delivery method collapsed into two groups (Vaginal vs Caesarean) due to low cell frequencies; ^‡^ Estimates calculated were exponentially small due to low cell frequencies. Underlying assumptions of independence of observations (Durbin-Watson (9, 141) = 1.196) and multicollinearity (mean VIF = 1.14) were met, and no severe outliers were observed. Adjusted regression model = (F(32, 141) = 84.62, p <.001); pseudo R^2^ = 35.57 | | | | |

| Online Supplement Table 4 *Cohort Sample Adjusted Linear Regression Analyses of Infant Feeding Practices*^†^ *and Beliefs About Breastfeeding Using BAB Differential Scores* | | | | |
| --- | --- | --- | --- | --- |
|  | β coefficient | SE | p | 95% CI |
| Ethnicity* |  |  |  |  |
| Asian | 0.01 | 0.39 | .978 | -0.77, 0.79 |
| Black | 0.06 | 0.52 | .913 | -0.98, 1.09 |
| Mixed | -0.30 | 0.46 | .524 | -1.22, 0.63 |
| Other | 0.46 | 0.54 | .398 | -0.62, 1.54 |
| Marital Status^‡^ |  |  |  |  |
| Married / Civil Partnership | 1.37 | 0.77 | .080 | -0.17, 2.90 |
| Cohabiting with Partner | 1.40 | 0.78 | .079 | -0.16, 2.96 |
| Partnered, not cohabiting | 1.13 | 1.36 | .409 | -1.57, 3.82 |
| Maternal Age | 0.01 | 0.03 | .758 | -0.05, 0.07 |
| Socioeconomic status | 0.02 | 0.05 | .656 | -0.08, 0.13 |
| Previous feeding | 0.07 | 0.02 | .001* | 0.03, 0.12 |
| Depression | 0.01 | 0.04 | .796 | -0.08, 0.10 |
| Anxiety | -0.07 | 0.06 | .221 | -0.18, 0.04 |
| Delivery^#^ |  |  |  |  |
| Vaginal assisted | 0.56 | 0.35 | .113 | -0.13, 1.25 |
| Caesarean Planned | -0.03 | 0.28 | .918 | -0.59, 0.53 |
| Caesarean Unplanned / Emergency | 0.24 | 0.38 | .526 | -0.52, 1.01 |
| BAB Differential | 0.02 | 0.03 | .600 | -0.04, 0.07 |
| _cons | -2.57 | 1.34 | .058 | -5.24, 0.08 |
| ^†^Standardized infant feeding scores (z-scores) used to correct for skew (χ^2^ (16) = 31.99, p = .010) and kurtosis (χ^2^ (1)= 7.56, p = .006). *‘White’ as reference base category; ^‡^‘Single’ as reference category; ^#^Category ‘Vaginal unassisted’ as reference base category. Underlying assumptions of independence of observations (Durbin-Watson (17, 97) = 1.04), Heteroscedasticity (χ^2^ (81) = 91.59, p = .198) and multicollinearity (mean VIF = 2.79) were met. Adjusted regression model = (F (16,80) = 2.11, p = .016); adjusted R^2^ = 15.56. | | | | |

| Online Supplement Table 5 *Adjusted Multinomial Regression Analyses of Breastfeeding Experiences and Beliefs About Breastfeeding Among Online and Cohort Samples Using BAB Differential Scores* | | | | | |
| --- | --- | --- | --- | --- | --- |
| Experience by Group | | RR | SE | p | 95% CI |
| Much More Negative | |  |  |  |  |
| Online | BAB differential | 0.83 | 0.04 | <.001* | 0.76, 0.92 |
|  | _cons | 0.14 | 0.09 | .003 | 0.04, 0.50 |
| Cohort | BAB Differential | 0.72 | 0.06 | <.001* | 0.62, 0.83 |
|  | _cons | 0.16 | 0.13 | .021 | 0.03, 0.75 |
| A Little More Negative | |  |  |  |  |
| Online | BAB differential | 0.98 | 0.05 | .717 | 0.90, 1.08 |
|  | _cons | 0.29 | 0.19 | .058 | 0.08, 1.04 |
| Cohort | BAB Differential | 0.93 | 0.05 | .167 | 0.83, 1.03 |
|  | _cons | 0.47 | 0.27 | .183 | 0.15, 1.43 |
| A Little More Positive | |  |  |  |  |
| Online | BAB differential | 1.07 | 0.05 | .178 | 0.97, 1.18 |
|  | _cons | 0.19 | 0.14 | .023 | 0.04, 0.80 |
| Cohort | BAB Differential | 1.00 | 0.05 | .929 | 0.91, 1.10 |
|  | _cons | 0.51 | 0.29 | .238 | 0.17, 1.55 |
| Much More Positive | |  |  |  |  |
| Online | BAB differential | 1.20 | 0.06 | <.001* | 1.09, 1.32 |
|  | _cons | 0.50 | 0.35 | .318 | 0.13, 1.96 |
| Cohort | BAB Differential | 1.18 | 0.06 | .002* | 1.06, 1.30 |
|  | _cons | 0.26 | 0.16 | .026 | 0.08, 0.85 |
| Base Outcome = “My experience with breastfeeding so far has been As I Expected”; RR= Relative Risk; SE= Standard Error; p = p value significant at α= .05; CI = Confidence Interval. Analyses were adjusted for delivery method, maternal distress (Online Sample), maternal depression and maternal anxiety (Cohort Sample). Online Sample underlying assumptions of independence of observations (Durbin-Watson (6, 277) = 1.89) and multicollinearity (mean VIF = 1.17) were met. Adjusted regression model = (F(20, 277) = 124.75, p <.001); pseud R^2^ = 14.11. Cohort Sample underlying assumptions of independence of observations (Durbin-Watson (7, 250) = 1.79) and multicollinearity (mean VIF = 1.67) were met. Adjusted regression models = (F(24, 250) = 112.44, p <.001); pseudo R^2^= 14.47. | | | | | |
